# P-111. Promising Efficacy of Nasal-Spraying *Bacillus* Spore probiotics for Supportive Treatment of Pneumonia in Children with RSV and Bacterial Co-infections

**DOI:** 10.1093/ofid/ofae631.318

**Published:** 2025-01-29

**Authors:** Thuy Thi Bich Phung, Anh Thi Van Nguyen, Huyen Thi Bui, Anh Hoa Nguyen, Hang Minh Nguyen, Hanh Thi Hong Le, Hoa Thi Le

**Affiliations:** Vietnam National Children's Hospital, Ha Noi, Ha Noi, Vietnam; ANABIO R&D Ltd., Hanoi, Ha Noi, Vietnam; ANABIO R&D Ltd., Hanoi, Ha Noi, Vietnam; LiveSpo Pharma Ltd. Hanoi, Vietnam, Ha Noi, Ha Noi, Vietnam; Vietnam National Children's Hospital, Ha Noi, Ha Noi, Vietnam; Vietnam National Children's Hospital, Ha Noi, Ha Noi, Vietnam; Vietnam National Children's Hospital, Ha Noi, Ha Noi, Vietnam

## Abstract

**Background:**

: Respiratory syncytial virus (RSV) and bacterial co-infections are primary cause of pneumonia. Recently, nasal-spraying *Bacillus* spore probiotics (LiveSpo NAVAX containing ≥ 5 billion/5mL *B. subtilis* and *B. clausii* spores) have demonstrated its effectiveness in supporting the treatment of acute respiratory tract infections caused by RSV and Influenza virus^1,2^.

**Methods:**

A randomized, double-blind, controlled clinical trial was conducted to evaluate the efficacy of LiveSpo NAVAX in the supportive treatment of severe pneumonia due to RSV and bacterial co-infections. A total of 120 eligible pediatric patients were randomly assigned to two groups: the Control group received 0.9% NaCl physiological saline, and the probiotic-intervention group (Navax group) received LiveSpo NAVAX three times a day.

**Results:**

At day 3, the Navax group demonstrated significant reductions in both RSV and bacterial co-infection 15.7 and 40.8-fold greater than those observed in the control group. Notably, *H. influenzae* and *S. pneumoniae* were reduced by 22.8 and 17.2 folds, respectively. Additionally, the Navax group exhibited significant decreases in pro-inflammatory cytokines (IL-6, IL-8, TNF-α), with levels reduced by 1.4 - 3.6 folds, while also enhancing the immune system (IgA) by over 67.04%. Furthermore, the 16S rRNA metagenomic analysis revealed the restoration of nasal microbiota, with an increase in the density of beneficial genus such as *Corynebacterium, Bacillus* and a reduction in the density of harmful genus including *Streptococcus*, *Haemophilus*, and *Moraxella*.

**Conclusion:**

Our data demonstrate that nasal-spray *Bacillus* spore probiotics supported the treatment of pneumonia in children by reducing RSV, co-infection bacterial loads, and pro-inflammatory cytokines.

**Disclosures:**

**All Authors**: No reported disclosures

